# GTDB release 10: a complete and systematic taxonomy for 715 230 bacterial and 17 245 archaeal genomes

**DOI:** 10.1093/nar/gkaf1040

**Published:** 2025-10-22

**Authors:** Donovan H Parks, Pierre-Alain Chaumeil, Aaron J Mussig, Christian Rinke, Maria Chuvochina, Philip Hugenholtz

**Affiliations:** Australian Centre for Ecogenomics, School of Chemistry and Molecular Biosciences, The University of Queensland, Brisbane, Queensland 4072, Australia; Center for Microbial Communities, Department of Chemistry and Bioscience, Aalborg University, Aalborg 9220, Denmark; Australian Centre for Ecogenomics, School of Chemistry and Molecular Biosciences, The University of Queensland, Brisbane, Queensland 4072, Australia; Center for Microbial Communities, Department of Chemistry and Bioscience, Aalborg University, Aalborg 9220, Denmark; Australian Centre for Ecogenomics, School of Chemistry and Molecular Biosciences, The University of Queensland, Brisbane, Queensland 4072, Australia; Department of Microbiology, University of Innsbruck, Innsbruck 6020, Austria; Center for Microbial Communities, Department of Chemistry and Bioscience, Aalborg University, Aalborg 9220, Denmark; Leibniz Institute DSMZ—German Collection of Microorganisms and Cell Cultures, Braunschweig 38124, Germany; Australian Centre for Ecogenomics, School of Chemistry and Molecular Biosciences, The University of Queensland, Brisbane, Queensland 4072, Australia

## Abstract

The Genome Taxonomy Database (GTDB; https://gtdb.ecogenomic.org) provides a phylogenetically consistent and rank normalized genome-based taxonomy for prokaryotic genomes sourced from the NCBI Assembly database. GTDB release 10 (R10-RS226) spans 715 230 bacterial and 17 245 archaeal genomes organized into 136 646 bacterial and 6968 archaeal species clusters. Fewer new major branches of prokaryotic life are being discovered with each release of GTDB, suggesting that we are beginning to saturate readily discoverable microbial diversity through culture-independent analyses. However, species discovery continues unabated as >95% of bacterial and archaeal species remain to be genomically elucidated based on conservative projections. We present additions to the GTDB website, methodological improvements, policy changes, notable nomenclatural updates, and user applications. We conclude with a summary of future plans for the resource including a fungal taxonomy and a nomenclatural extension to classify pathogens.

## Graphical Abstract

**Graphical Abstract:**
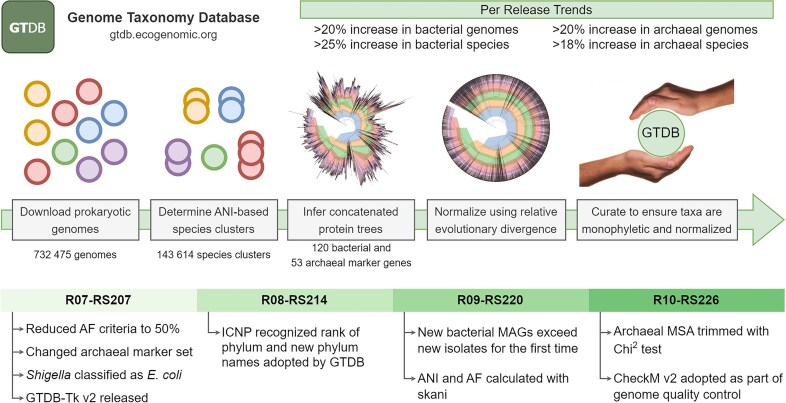

## Introduction

The Genome Taxonomy Database (GTDB) provides a phylogenetically consistent taxonomy for *Bacteria* and *Archaea* that uses average nucleotide identity (ANI) to operationally delineate species [1] and relative evolutionary divergence (RED) applied to concatenated marker gene trees to define higher taxa [2, 3]. These criteria ensure all genomes are categorized from species to domain and provide an objective phylogeny-based definition for taxonomic ranks across the bacterial and archaeal domains. This permits the systematic taxonomic classification of isolate genomes, single amplified genomes (SAGs), and the millions of metagenome-assembled genomes (MAGs) being obtained from environmental, clinical, and engineered sources [4–6]. GTDB is supported by GTDB-Tk, a stand-alone tool that provides classifications of user genomes against the GTDB taxonomy [7]. GTDB-Tk was updated in 2022 to use a divide-and-conquer approach that better scales with database growth [8]. There are ongoing efforts to demonstrate or improve the integrity of the GTDB such as a sensitivity analysis of the taxonomy to genome contamination [9] and formal proposal of legacy Latin names introduced during early curation cycles [10].

The latest version of the GTDB is R10-RS226, released in April 2025. This is the 10th release (i.e. R10) of the GTDB since its inception in November 2017. R10-RS226 covers genomes in the NCBI (National Center for Biotechnology Information) Assembly database as of September 2024 when RefSeq 226 (i.e. RS226) was released [11]. This manuscript explores the growth of the GTDB from inception together with website, methodology, policy, and major taxonomic and nomenclatural updates that have occurred since R06-RS202. Updates from inception to R06-RS202 have been previously reported [12].

## Resource content

### Growth of GTDB

**Figure 1.**
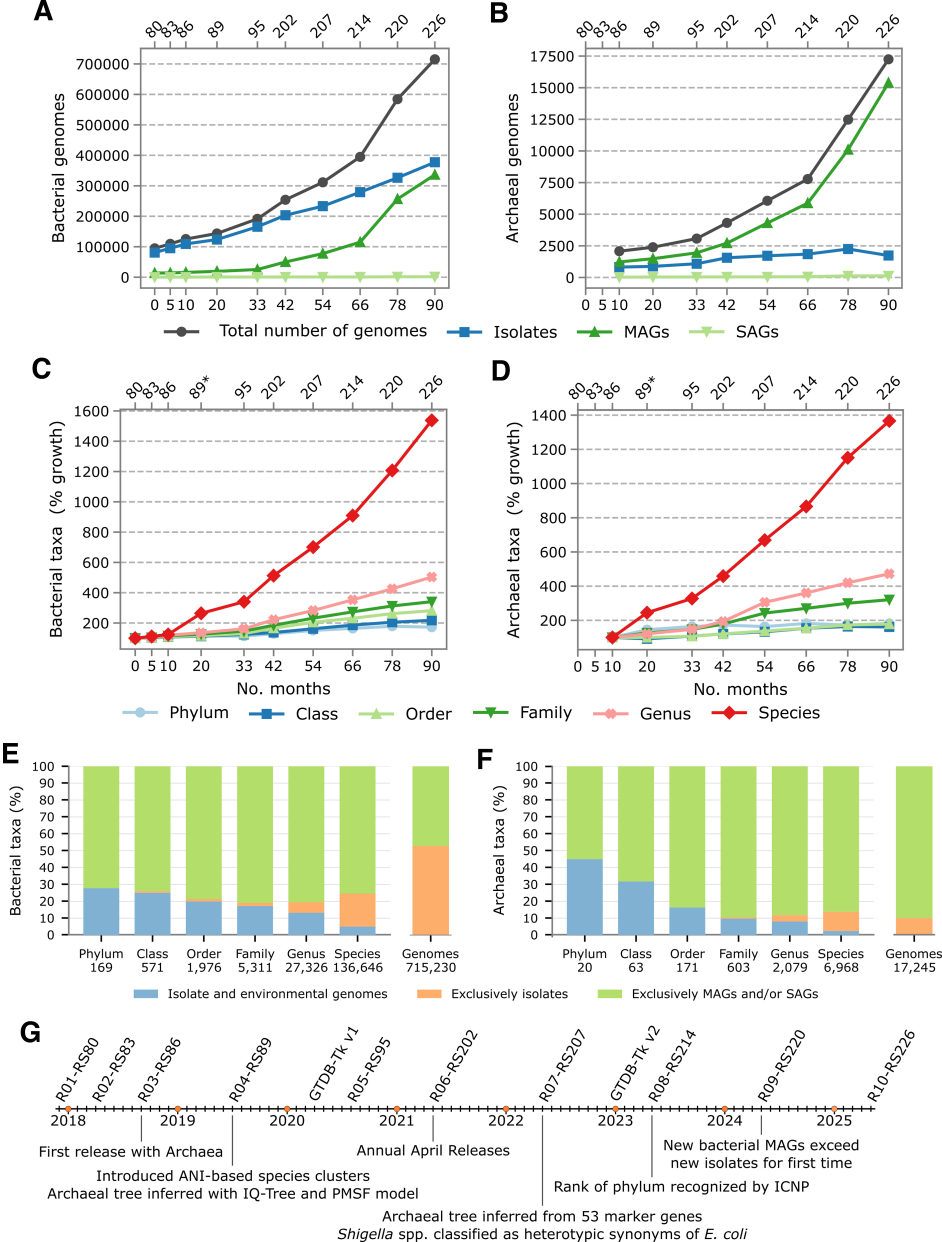
Growth (**A–F**) and timeline of major events (**G**) since inception of GTDB in November 2017. (**A, B**) Number of bacterial and archaeal isolates, MAGs, and SAGs in the GTDB along with the total number of genomes. The top *x*-axis shows GTDB release numbers and the bottom *x*-axis indicates months since inception. (**C, D**) Percent growth in the number of bacterial and archaeal taxa in the GTDB since inception. (**E, F**) Proportion of bacterial and archaeal taxa at each taxonomic rank in GTDB R10-RS226 comprised exclusively of environmental genomes (MAGs and/or SAGs), exclusively of isolates, or containing both isolate and environmental genomes. For comparison, the overall proportion of isolate and environmental genomes is shown in the right bar plot.

The number of genomes in the GTDB has grown by over 22% with each release since 2021, with a notable increase of 48% between R08-RS214 in 2023 and R09-RS220 in 2024 (Fig. 1A and B, and Supplementary Table S1). A substantial portion of the 194 150 genomes introduced in R09-RS220 can be attributed to the inclusion of 52 605 isolate genomes and MAGs from the Mouse Gastrointestinal Bacteria Catalogue (MGBC) [13] and 16 626 MAGs from the Integrated Mouse Gut Metagenome Catalog (iMGMC) [14]. R10-RS226 includes 135 616 new genomes including 9107 MAGs from the Soil Microbial Dark Matter Catalogue (SMAG) [5]. While large genome catalogues account for an appreciable number of new genomes, the growth of GTDB is largely the result of hundreds of smaller studies contributing genomes to the NCBI Assembly database each year (Supplementary Table S2). Notably, the number of new bacterial MAGs exceeded the number of new bacterial isolates starting with R09-RS220 in 2024 (141 593 MAGs versus 47 276 isolates) and this trend has continued in R10-RS226 (79 790 MAGs versus 50 958 isolates). By contrast, in the archaeal dataset, MAGs have exceeded isolate genomes in terms of both absolute numbers and additions in each release since their introduction in R03-RS86 in August 2018 [3], likely due to greater technical hurdles in culturing *Archaea*.

Delineation of species clusters by ANI was introduced in R04-RS89 in June 2019 (Fig. 1G) [1]. The number of bacterial and archaeal species clusters has increased by an average of 34.4% and 33.5%, respectively, in the six subsequent releases (Fig. 1C and D, and Table 1). Growth in the number of taxa at the rank of genus, family, and order has been more modest, and the rate of growth has generally decreased starting with R07-RS207 in April 2022. Notably, the number of archaeal classes decreased for the first time in R10-RS226 from 64 to 63 due to changes in taxonomic opinion and the number of bacterial classes increased by only 6.1%, much lower than in previous releases (average 12.7%). The number of archaeal phyla has varied between 18 and 20 since R05-RS95 in 2020, suggesting that the discovery of new major lineages through MAG reconstruction has likely reached saturation. This conclusion is supported by a recent, large-scale effort to recover MAGs from the rare biosphere that discovered six new bacterial phyla, but no new archaeal phyla [15]. The number of bacterial phyla had been gradually increasing until R09-RS220 but then decreased from 175 to 169 in R10-RS226 due to merging of several stably associated phyla, suggesting that we are also beginning to saturate readily discoverable higher level bacterial diversity. By contrast, there is a steady upward trajectory of new prokaryotic species consistent with a conservative global estimate [albeit 16S rRNA (ribosomal RNA) gene-based] of 2.2–4.3 million prokaryotic species [16], i.e. R10-RS226 represents only 3.3%–6.5% of this species estimate range.

**Table 1.** Changes in the number of taxa across GTDB releases since introduction of ANI-based species clusters in R04-RS89 (June 2019)

Rank	Domain	Average change (%)	Minimum change (%)	Maximum change (%)
Species	*Bacteria*	34.4	27.4	50.7
Species	*Archaea*	33.5	18.7	45.9
Genus	*Bacteria*	24.6	18.2	37.1
Genus	*Archaea*	26.3	12.6	57.9
Family	*Bacteria*	18.1	9.1	26.5
Family	*Archaea*	17.1	6.9	35.7
Order	*Bacteria*	16.1	7.4	26.8
Order	*Archaea*	10.2	3.0	13.8
Class	*Bacteria*	11.6	6.1	18.1
Class	*Archaea*	9.9	−1.6	16.7
Phylum	*Bacteria*	7.4	−3.4	16.5
Phylum	*Archaea*	4.0	−5.3	12.5

MAGs account for the majority of bacterial and archaeal taxonomic diversity in GTDB R10-RS226 (Fig. 1E and F, and Supplementary Table S1). Over 70% of bacterial taxa, regardless of rank, are exclusively environmental sequences despite MAGs (336 367 genomes) and SAGs (1470 genomes) representing only 47.2% of the 715 230 bacterial genomes. Similarly, over 50% of all archaeal taxa, regardless of rank, consist exclusively of MAGs and/or SAGs with over 80% of archaeal species, genera, families, and orders lacking an available cultured representative. In contrast to *Bacteria*, nearly 90% of all archaeal genomes are MAGs.

### Changes to the GTDB website

**Figure 2.**
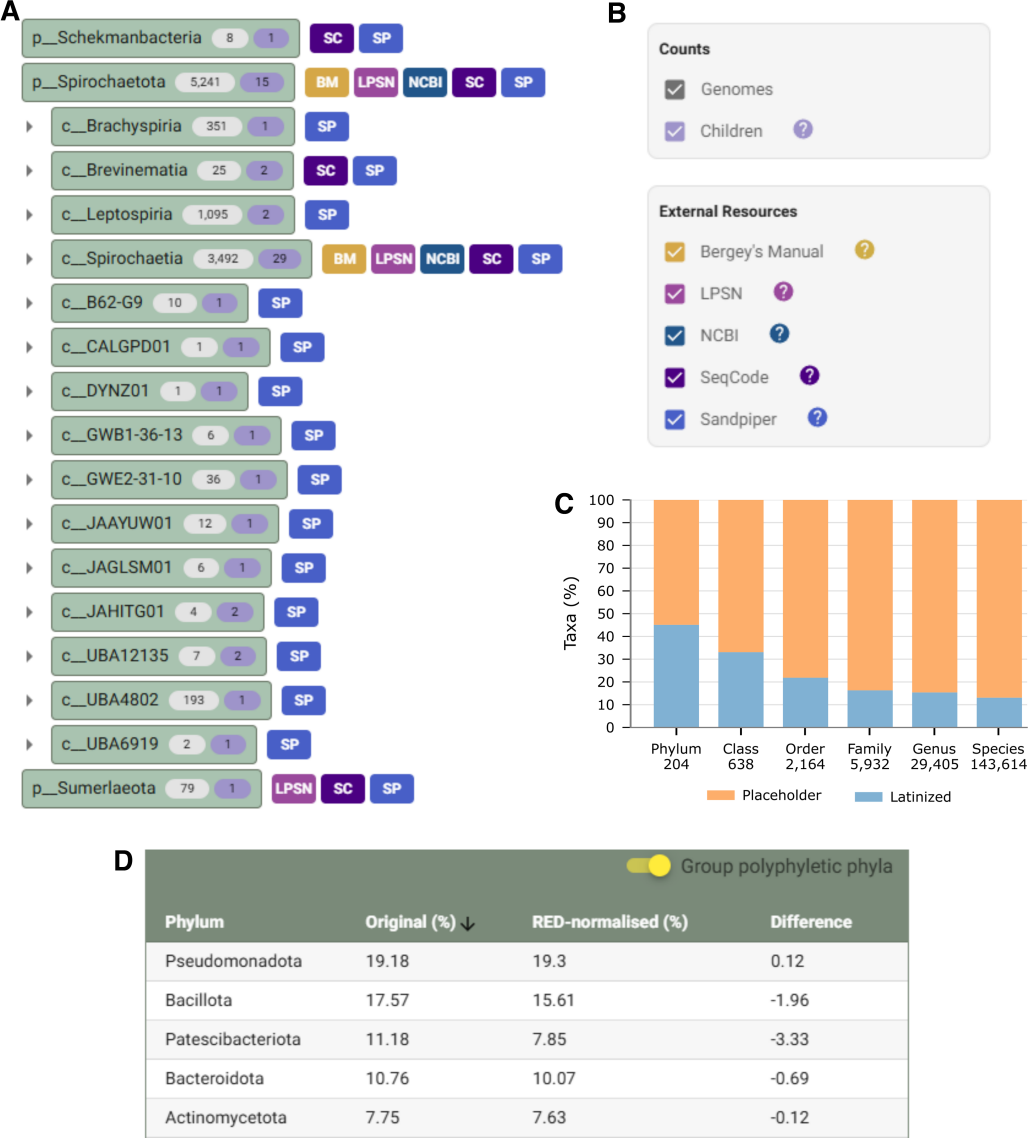
Recent updates to the GTDB website. (**A**) Addition of user-selectable links to external resources in the Taxonomy Tree view and taxon counts for descendant genomes and children taxa. (**B**) Control panels allowing users to select external links or taxon counts to be displayed in the Taxonomy Tree view. (**C**) Plot provided with each GTDB release that indicates the number of taxa at each rank with placeholder or Latin names. (**D**) Table provided with each GTDB release that indicates Faith’s Phylogenetic Diversity for each phylum on both the original GTDB reference trees and the RED-normalized GTDB reference trees.

Additional features and improvements have been made to the GTDB website since our last report [12]. We now link GTDB taxa to several external resources through the Taxonomy Tree view (Fig. 2A). This includes links to prominent nomenclatural and taxonomic resources such as Bergey’s Manual [17], List of Prokaryotic names with Standing in Nomenclature (LPSN) [18], SeqCode (SC) [19], and NCBI Taxonomy pages [20]. We have also provided links to Sandpiper [21], an exploratory resource showing the geographic and environmental distribution of taxa in metagenomic datasets, and are open to including other resources in the future. In addition to displaying links to external resources, the Taxonomy Tree view can display the number of genomes or children taxa. For example, this permits one to quickly establish that the phylum *Spirochaetota* contains 15 classes and that the GTDB contains 5241 genomes assigned to this phylum. The icons providing links to external resources or displaying taxon metadata are all optional, allowing users to tailor this view to suit their requirements (Fig. 2B). The sorting of taxa in the Taxonomy Tree view has also been improved to display the type strain of the species first and Latin names before placeholder names. GTDB data are now available from both the University of Queensland in Australia and Aalborg University in Denmark to facilitate downloading these data in different regions (gtdb.ecogenomic.org/downloads).

We have added new resource-wide statistics for each release that provide further insights into nomenclatural and biodiversity aspects of the database. This includes a table and bar plot indicating the number of GTDB taxa at each rank that have Latin or placeholder names (Fig. 2C). For example, in GTDB R10-RS226, 86.9% of bacterial and archaeal species have placeholder names, illustrating the wealth of diversity that remains to be formally described. Notably, 362 GTDB taxa with placeholder names have one or more subordinate taxa with Latin names indicating that only 0.3% of current placeholder names could be formally named based on existing Latin names. The phylogenetic diversity of each phylum is now tabulated for each release with the option to group phyla that are polyphyletic in the GTDB reference tree, but for which there is alternate evidence supporting their monophyly (Fig. 2D). Phylogenetic diversity is calculated over both the original reference trees for each domain and RED-normalized trees. In R10-RS226, four bacterial and three archaeal phyla account for over half of the phylogenetic diversity in their respective domains: *Pseudomonadota* (19.2%), *Bacillota* (17.6%), *Patescibacteriota* (11.2%), and *Bacteroidota* (10.8%); and *Nanobdellota* (28.1%), *Thermoproteota* (18.0%), and *Thermoplasmatota* (12.3%). Finally, we have added a toggle to the GTDB Statistics page that changes all plots to colour vision deficiency-safe palettes.

The Genome pages and Advanced Search page have also been updated. Genome pages now provide CheckM v1 [22] and CheckM v2 [23] statistics, with quality estimates from both versions used to establish the set of genome assemblies included in GTDB (see below). The Advanced Search page now allows the generation of a shell script that can be used to download different data files (e.g. genomic FASTA, GFF, or CDS files) for all genomes present in the search results using either curl or the NCBI datasets tool.

## Resource methodology and policies

### Methodological updates

Methodological changes have been made to improve the computational efficiency and quality of the resource since our last report [12]. Regarding quality control (QC), the maximum number of permissible contigs in a genome was raised in R08-RS214 from 1000 to 2000 to be consistent with the filtering criterion used by RefSeq. More substantively, CheckM v2 genome quality estimates have been adopted as part of our QC criteria beginning with R10-RS226. Specifically, a genome now must have completeness ≥50%, contamination <5%, and quality (completeness − 5 × contamination) ≥50% according to both the CheckM v1 and v2 estimates. The exception to this new criterion is that a genome comprising <10 contigs passes QC if the genome quality estimates are satisfied by either CheckM v1 or v2. Adoption of CheckM v2 quality estimates and exception for genomes with <10 contigs resulted in 12 214 (11.1% increase) additional genomes failing QC and only 178 (0.023% increase) additional genomes passing QC, demonstrating that these changes largely result in higher stringency.

With regard to genome clustering, we reduced the alignment fraction (AF) criterion used to assign genomes to a species representative from 65% to 50% in R07-RS207. This change was made to accommodate the growing number of MAGs in GTDB, many of which are incomplete. Comparing partial genomes can result in artificially low AF values. The impact of this change was evaluated on R06-RS202 where 274 species representatives would have been merged if the AF criterion was lowered to 50%. These genome pairs had an average ANI of 97.5%, indicating that if complete genomes were available they would likely be assigned to the same species. As expected, these genomes had an average completeness of only 71%.

In terms of computational efficiency, we replaced FastANI [24] with skani [25] for calculating ANI and AF values in R09-RS220. Skani is an order of magnitude faster than FastANI and provides more accurate results when applied to fragmented, incomplete genomes as is often the case for MAGs [25]. We validated this transition by comparing skani to FastANI results across 11 million intra-genus genome comparisons. The mean difference in ANI values was 0.077%, with skani producing slightly higher values on average. Concomitantly, skani produces slightly lower AF values on average with a mean difference of 1.68%. As further validation, we reprocessed the GTDB R08-R214 genomes using skani and found that 316 054 of 317 504 genomes (99.54%) were assigned to the same representative genome. Furthermore, the set of new species representatives selected in R08-R214 were nearly identical (99.92% overlap) when using FastANI or skani. These results illustrate that changing from FastANI to skani had a marginal impact on GTDB species assignments, while reducing computational burden.

The methodology used to infer the archaeal reference tree has been revised to reflect current best practices. The marker set used for phylogenetic inference was changed from the 122 archaeal markers proposed by Parks *et al.* [26] to a set of 53 markers adopted from Dombrowski *et al.* [27] beginning with R07-RS207. This change improved marker coverage across archaeal lineages and the robustness of the genome phylogeny based on average bootstrap support for nodes (84.7% versus 88.6%). Another focus was on improving the modelling of variations in substitution rates among amino acids, termed across-site compositional heterogeneity [28], and differences in substitution patterns between lineages, known as branch-wise compositional heterogeneity [29]. Across-site compositional heterogeneity arises from differing physicochemical properties of amino acids, such as charge and size, which influence protein structure and function and result in varying substitution rates. We implemented a protein mixture model (PSMF approximation of the C10 model) starting with R04-RS89, to better address these across-site heterogeneities in the archaeal phylogeny. Traditional substitution models often assume a uniform rate of evolution across all amino acid sites, and this simplification can result in molecular convergence between taxa being misinterpreted as evidence of close evolutionary relationships [30]. By contrast, mixture models such as the C10-C60 models [31] partition protein sequences into multiple ‘categories’, each characterized by its own distinct amino acid substitution rates. Mixture models have been shown to provide a better fit for biological data and to more effectively account for multiple substitutions per site (saturation), which in turn minimizes artifacts like long-branch attraction [28, 32].

Branch-wise compositional heterogeneity can arise through shared environmental adaptation of evolutionarily distant lineages. Well-documented examples include thermophiles, which have evolved to preferentially use thermostable amino acids [33], or halophiles that have adapted to high-salt environments [34]. To account for these differences in substitution patterns between lineages, we removed the top 20% most compositionally biased sites from the archaeal alignment, ranked according to chi-square scores by the alignment_pruner.pl script [29], starting with R10-RS226. This approach resolved, for example, the placement of the recently proposed genera *Njordarchaeum* and *Panguiarchaeum* in the class *Njordarchaeia* of the phylum *Asgardarchaeota* [29], and of *Korarchaeota* as a sister phylum-level lineage to *Thermoproteota* and *Asgardarchaeota*. Note that, these approaches currently do not computationally scale to the bacterial reference tree.

### Nomenclatural updates and challenges

Since our previous report on GTDB [12], a substantial number of changes have occurred in the field of prokaryotic nomenclature. These include official recognition of the rank of phylum in the International Code of Nomenclature of Prokaryotes (ICNP) [35], followed by an initial validation of 42 phylum names [36]. The replacement of several commonly used but not validly published names with substantively different valid names, e.g.*‘Proteobacteria’* to *Pseudomonadota* and *‘Firmicutes’* to *Bacillota*, was criticized [37]; however, the NCBI Taxonomy [20] and LPSN [18] have adopted the new names, which is reflected in recent publications (e.g. >11 000 results for *Pseudomonadota* and *Bacillota* in Google Scholar June 2025). This created a one-off abrupt and widespread change of phylum names in GTDB (2023 release R08-RS214; Fig. 1G), which we expect will not occur again since all commonly used taxonomic ranks for prokaryotes are now formalized. Most recently, this includes the rank of kingdom that sits between domain and phylum [38]. There is increasing phylogenetic evidence that bacterial diversity has a primary bifurcation between monoderms and diderms [39–41], which can now be formally classified as the kingdoms *Bacillati* (formerly ‘*Terrabacteria*’) and *Pseudomonadati* (formerly ‘*Gracilicutes*’). In the archaeal domain, the three kingdoms *Methanobacteriati*, *Nanobdellati*, and *Thermoproteati* were proposed with the intention of reflecting the earlier division into *‘Euryarchaeota’*, ‘DPANN superphylum’, and ‘TACK superphylum’, respectively [38]. While *Nanobdellati* could be implemented in GTDB, the former *‘Euryarchaeota’* do not represent a monophyletic clade and the former ‘TACK superphylum’ is represented by the phylum *Thermoproteota*, based on RED normalization. The rank of kingdom has not yet been incorporated into the GTDB taxonomy, but its inclusion is being investigated (see the ‘Future Plans’ section).

Another significant development is the establishment of a new nomenclatural code based on genome sequence data, the SeqCode [19], which is now a linked external resource in the Taxonomy Tree view (Fig. 2A). This is an important development for microbiologists as it allows formal naming of the thousands of as-yet-uncultured taxa that comprise most of the diversity in GTDB [42]. Previously, we reported that Latin names are no longer proposed by GTDB curators unless there is an associated publication with taxon descriptions [12]. Until recently, several hundred *ad hoc* Latin names were still present in GTDB from earlier releases (flagged in Taxonomy Tree view with #). Most of these have now been formally proposed by leveraging both the ICNP and SeqCode [10].

GTDB was originally established as a taxonomic framework to systematically classify the vast diversity of uncultured prokaryotes found in nature [2]. It was not intended for identification of pathogens, which are often distinguished by virulence factors that vary at the subspecies level and can be horizontally transferred [43]. A case in point was the erroneous identification of anthrax by a South African public health laboratory based on classification of two meat-borne isolates as *Bacillus anthracis* using GTDB-Tk [44]. On further investigation, these isolates were found to lack the plasmids encoding anthrax toxin (pXO1) and capsule (pXO2); pXO plasmids are mostly associated with a clonal expansion in *B. anthracis*, although they are also occasionally found in neighbouring species [44]. Another example is the *E. coli*/*Shigella* complex that we accommodated in earlier releases of GTDB by subdividing the ANI-based *E. coli* to recognize *Shigella* species resulting in new names such as ‘*Escherichia flexneri’* and ‘*E. dysenteriae’*. This was a suboptimal solution that was subsequently reversed by reclassifying all *Shigella* species as heterotypic synonyms of *E. coli* in GTDB R07-RS207 [45]. These examples highlight the current inadequacy of GTDB for classifying pathogens and the need for a classification system that divorces strictly vertical evolution (the GTDB framework) from horizontally acquired traits often seen at the subspecies level (see the ‘Future Plans’ section).

### Policy updates

After initial exploration of six and nine monthly releases, we have settled into an annual release in April of each year beginning with R06-RS202 (Fig. 1G). This provides users with certainty on the timeframe of new releases and is sufficiently frequent to keep up with the availability of new genome assemblies. We recognize SeqCode (discussed above) meaning that ICNP and SeqCode names compete for priority in the GTDB taxonomy. Two important phylum-level examples are *Asgardarchaeota* (validly published under SeqCode-15 June 2024) [46] used instead of its later synonym *Promethearchaeota* (valid under ICNP-5 July 2024) [47] and *Patescibacteriota* (valid under SeqCode-19 November 2024) [48] used instead of its later synonym *Minisyncoccota* (valid under ICNP-7 February 2025) [49]. However, in certain circumstances we use easily discoverable valid later synonyms (in either nomenclature) if they are more widely used by the research community. For example, the names *Bacillales* and *Caryophanales* are both validly published under the ICNP, but we prefer the later synonym *Bacillales* because it has been used much more frequently than *Caryophanales* in publications (>420×; Google Scholar, June 2025) and is flanked by ranks with names sharing the same generic stem, i.e.*Bacilli* and *Bacillaceae*. Note that this case does not apply to, for example, the phylum *Pseudomonadota* and ‘*Proteobacteria*’ because the latter name is not validly published under either code. Where available, we use standardized NCBI whole genome shotgun project identifiers (e.g. JAADFP01) as placeholder names for new taxa delineated during the curation process with the exception of taxa that include cultured representatives. In these cases, we use culture collection strain identifiers. For example, the family DSM-23947 contains *Oikeobacillus pervagus* DSM 23947^T^, which alerts users to the presence of cultured representatives in a GTDB-defined taxon that could be named after the isolate, i.e. ‘*Oikeobacillaceae’*. In 2023, we established a Scientific Advisory Board comprising nine globally recognized experts in taxonomy, nomenclature, and phylogenetics to help guide the direction of the resource.

## GTDB user applications

GTDB has developed a large and varied user base, attesting to the continued uptake and utility of the resource. As of August 2025, GTDB-Tk has been downloaded almost 300 000 times, and the website receives ∼15 000 unique visitors per month. User applications can be broadly divided into three categories:

### Third party software and databases

The GTDB taxonomy has been incorporated into a wide range of web services, bioinformatic tools, and genome reference databases. A non-exhaustive list is provided in Table 2. Notable recent applications include GARNET [50] and Sandpiper [21]. The GARNET (Gtdb Acquire RNa with Environmental Temperatures) database and associated machine-learning models derived from this database were established to improve functional predictions of structural RNAs. GTDB was adopted as the taxonomic anchor for GARNET as it provides greatly expanded RNA sequence diversity and a framework for linking sequences to experimental data [50]. Sandpiper is an interactive web-based tool that allows users to visualize the geographic distribution and relative abundance of GTDB-defined taxa based on their presence in metagenomic datasets [21]. This resource has recently been added as a user-selectable external link in the Taxonomy Tree view (Fig. 2A).

**Table 2.** Examples of third-party resources that use or incorporate the GTDB taxonomy

Resource	Description	URL	Reference
AutoMLST2	Genome taxonomic classifier that incorporates GTDB	automlst2.ziemertlab.com	[74]
AnnoTree	Web server for visualizing annotations on a phylogenetic tree that incorporates GTDB	annotree.uwaterloo.ca/annotree	[75]
AnnoView	Web server for visualizing gene neighborhoods that incorporates GTDB	annoview.uwaterloo.ca/annoview	[76]
BakRep	Genome repository with enriched metadata, uses GTDB taxonomy	bakrep.computational.bio	[77]
Bactopia	Pipeline for analysis of prokaryotic genomes, including classification with GTDB-Tk	github.com/bactopia/bactopia	[78]
Ganon	Metagenome profiler that incorporates GTDB	github.com/pirovc/ganon	[79]
GARNET	Database for RNA structural and functional analysis anchored to GTDB	github.com/Doudna-lab/GARNET_DL	[50]
Genomes OnLine Database	Catalogues and manages information related to (meta)genomic sequence projects; provides GTDB-Tk classifications for isolate genomes	gold.jgi.doe.gov	[80]
Greengenes2	16S rRNA database that incorporates GTDB, with QIIME2 plugin	github.com/biocore/q2-greengenes2	[81]
GlobDB	Genome database that includes GTDB species representatives	globdb.org	-
IDTAXA	16S rRNA classifier that incorporates GTDB	decipher.codes	[82]
IMG/MER	Online platform for annotation and analysis of microbial genome and microbiome datasets; provides GTDB-Tk classifications	img.jgi.doe.gov	[83]
iTOL	Web server for visualizing and manipulating phylogenetic trees with support for GTDB	itol.embl.de	[84]
KBase	Provides access to GTDB-Tk as a web service	kbase.us	[85]
KSGP	16S rRNA database for annotation of archaeal metabarcoding data that incorporates GTDB	ksgp.earlham.ac.uk	[86]
mOTUs	Metagenome profiler that incorporates GTDB	motus-db.org	[87]
Protologger	Genome description tool that uses GTDB-Tk to classify genomes	github.com/thh32/Protologger	[88]
Sandpiper	Provides geographic and ecological information for GTDB taxa	sandpiper.qut.edu.au	[21]
Sativa DBs	Provides 16S rRNA databases based on GTDB for DADA2	doi.org/10.17044/scilifelab. 14869077.v9	[89]
SeqCode	Repository for names proposed under the SeqCode that incorporates GTDB taxonomy	registry.seqco.de	[19]
SingleM	Metagenome profiler that incorporates GTDB	wwood.github.io/singlem	[21]
SPIRE	Catalogue of 1.16 million MAGs with GTDB taxonomic classifications	spire.embl.de	[4]
Sourmash	Metagenome profiler that incorporates GTDB	sourmash.readthedocs.io	[90]
Sylph	Metagenome profiler that incorporates GTDB	github.com/bluenote-1577/sylph	[91]

### Quantification of taxonomic diversity in MAG-based studies

An early and persistent use of the GTDB taxonomy is as a ‘yard stick’ of phylogenetic novelty in metagenomic studies that produce MAGs. Typically, the phylogenetic novelty of a MAG dataset is reported relative to a given release of the GTDB at species and higher taxonomic ranks. Notable recent examples include the SPIRE catalogue that encompasses MAGs from almost 100 000 global metagenomes [4] and the Microflora Danica catalogue that includes high-quality long read MAGs from Danish terrestrial habitats [51]. Another quantitative use of GTDB is a recent revision of 16S rRNA gene sequence identities to define taxonomic novelty. Rather than setting identity thresholds for ranks as previously proposed [52], 16S rRNA sequence identities were mapped to GTDB RED ranges for each rank [53]. This created overlapping windows of 16S rRNA sequence identities for adjacent ranks, providing greater flexibility for defining taxa.

### Facilitation of biological insights

GTDB has been instrumental to providing biological insights in a number of studies. For example, an as-yet-uncultured lineage of bacteria, TANB77, only recognized in the GTDB taxonomy, was found to be a gut microbiome biomarker of immune checkpoint blockade (ICB) response in a mouse model of lung cancer. A pilin-like protein specific to the TANB77 lineage enhanced responses to ICB therapy providing the basis for a new adjunct immunotherapy treatment [54]. A second example is the use of GTDB to select 1007 genomes representing most order-level lineages across the bacterial domain to perform an ancestral gene content inference [55] that when combined with machine learning provided a geological timescale for oxygen adaptation in *Bacteria* [56]. Balanced sampling of phylogenetic groups has been an ongoing obstacle in evolutionary studies due to lack of taxonomic consistency prior to the advent of the GTDB.

## Future plans

### Genome data not captured in GTDB

Currently GTDB obtains prokaryotic genomes from a single source, the NCBI Assembly database. This has two advantages: (i) the sequence and associated metadata is public and widely accessible via the International Nucleotide Sequence Database Collaboration (INSDC) [57] repositories and (ii) the data is in a uniform format facilitating release updates. However, several large genome collections from environmental sources have yet to be deposited in an INSDC repository and therefore are not represented in GTDB. These include SPIRE [4], GEM [58], GOMC [59], and TPMC [60]. The latter two catalogues have been deposited into the China National GeneBank Database [61] and China National Center for Bioinformatics [62], respectively, and hence may not be deposited into an INSDC repository. Therefore, we will explore the feasibility of adding one or both of these repositories as secondary sources of prokaryotic genomes as they are likely to comprise an increasingly large number of genomes not available via INSDC. Recently, we became aware of ∼11 000 genomes in the NCBI Assembly database that have not been incorporated into GTDB because they are taxonomically classified as metagenomes (Supplementary Table S3). We encourage submitters to update their genome metadata to indicate at least the domain of all genomes to ensure that these genomes are incorporated into GTDB in upcoming releases.

### Planned taxonomic extensions

With mounting evidence that the rank of kingdom makes both taxonomic and evolutionary sense [38, 56], we are exploring incorporation of this rank into the GTDB prokaryotic taxonomy. For *Bacteria*, this will require a more robust reference phylogeny than that afforded by a species representative tree inferred using the heuristic FastTree v2 method [63], which provides limited resolution of inter-phylum relationships. At the other end of the spectrum, we are exploring the feasibility of a nomenclatural extension system for pathogens that separates vertical evolution (domain to species) from horizontal/subspecies evolution of pathogenic traits. For example, *Bacillus anthracis* genomes that contain the pXO plasmids would be named *B. anthracis* gv. *anthrax* (gv., genovar) to indicate that they are likely pathogenic. This approach would also allow multiple extensions to be added if more than one pathogen classification system is widely used, as is the case for *E. coli* [43, 64], and it could be extended to non-pathogens with important transferable subspecies traits such as legume symbionts [65].

We have committed to extending GTDB to fungal genomes using the prokaryotic taxonomic framework as a template. This will include QC, ANI-based species delineation, concatenated marker gene phylogeny, and RED-normalized higher ranks. Elements of this approach have already been demonstrated including ANI-based fungal species [66] and a robust concatenated protein phylogeny for fungi [67]. With improving high throughput sequencing technologies, fungal genome sequencing is becoming commonplace and it is possible to obtain high quality fungal genomes from metagenomes, particularly using long read sequencing [68] and proximity ligation to link chromosomes [69], suggesting that the time has come for a comprehensive genome-based fungal taxonomy. We plan to make a beta version of the taxonomy available to fungal experts for initial evaluation and feedback.

### Concluding remarks

GTDB is a living resource and in addition to the future plans described above, version 3 of GTDB-Tk and a custom tool for predicting translation tables, gTranslate, are in development. Feedback is essential for GTDB to best serve the community and we encourage suggestions and comments on either the GTDB Forum (https://forum.gtdb.ecogenomic.org) or directly to the GTDB team (https://gtdb.ecogenomic.org/contact).

## Supplementary Material

gkaf1040_Supplemental_File

## Data Availability

The GTDB website can be accessed at https://gtdb.ecogenomic.org and data files for each GTDB release are available from Australia: https://data.ace.uq.edu.au/public/gtdb/data/releases Denmark: https://data.gtdb.aau.ecogenomic.org/releases GTDB builds upon existing public resources to provide an up-to-date taxonomic resource that reflects recently proposed taxa, changes in taxonomic opinion, and the wealth of publicly available genomes. The NCBI Taxonomy database (https://www.ncbi.nlm.nih.gov/taxonomy) [20] aids in the discovery of newly proposed taxa, provides initial species assignments, and specifies co-identical strain identifiers for genomes in the NCBI Assembly database. We use the NCBI Assembly database (https://www.ncbi.nlm.nih.gov/datasets/genome) [70] as the sole genome repository for the GTDB as it is a member of the INSDC [57], which also contains genomes submitted to DDBJ [71] and EMBL-EBI [72]. LPSN (https://lpsn.dsmz.de) [18] and SeqCode (https://registry.seqco.de) [19] are used as nomenclatural resources to establish co-identical strain identifiers for the type strains of species and subspecies, the types of higher-rank taxa, and the nomenclatural status of newly proposed or reclassified taxa. The Living Tree Project (https://imedea.uib-csic.es/mmg/ltp) [73] is used to classify 16S rRNA sequences and help resolve ambiguity regarding the correct classification of genomes.
