# Correction to ‘GTDB release 10: a complete and systematic taxonomy for 715 230 bacterial and 17 245 archaeal genomes’

**DOI:** 10.1093/nar/gkaf1458

**Published:** 2025-12-19

This is a correction to: Donovan H Parks, Pierre-Alain Chaumeil, Aaron J Mussig, Christian Rinke, Maria Chuvochina, Philip Hugenholtz, GTDB release 10: a complete and systematic taxonomy for 715 230 bacterial and 17 245 archaeal genomes, Nucleic Acids Research, 2025;, gkaf1040, https://doi.org/10.1093/nar/gkaf1040

Aalborg University was incorrectly listed under affiliations 1 and 4 during typesetting. This error has now been corrected.

This change does not affect the results, discussion and conclusions presented in the article. The published article has been updated.

