# Supplementary material for: GTDB release 10: a complete and systematic taxonomy for 715 230 bacterial and 17 245 archaeal genomes
Source: Nucleic Acids Res. 2025 Oct 22;54(D1):D743–54. doi: 10.1093/nar/gkaf1040 (PMC12807784; doi:10.1093/nar/gkaf1040)
Supplement: gkaf1040_Supplemental_File [file gkaf1040_supplemental_file.pdf]

GTDB release 10: a complete and systematic taxonomy for 715,230 bacterial and 17,245 archaeal genomes

Donovan H. Parks, Pierre-Alain Chaumeil, Aaron J. Mussig, Christian Rinke,
Maria Chuvochina, Philip Hugenholtz

Supplementary Table 1. Growth in number of genomes and taxa in the GTDB.

	R04-RS89	*R05-RS95*	*R06-RS202*	*R07-RS207*	*R08-RS214*	*R09-RS220*	*R10-RS226*
**Release date**	17-Jun-19	17-Jul-20	27-Apr-21	8-Apr-22	28-Apr-23	24-Apr-24	24-Apr-25
**Bacterial genomes**	143,512	191,527	254,090	311,480	394,932	584,382	715,230
Isolates	122,939	164,719	202,676	233,211	279,159	326,435	377,393
MAGs	19,880	26,107	50,669	77,521	114,984	256,577	336,367
SAGs	693	701	745	748	789	1,370	1,470
**Archaeal genomes**	2,392	3,073	4,316	6,062	7,777	12,477	17,245
Isolates	777	946	1,195	1,714	1,845	2249	1734
MAGs	1,574	2,079	3,068	4,304	5,874	10,102	15,385
SAGs	41	48	53	44	58	126	126
**Bacterial taxa**							
Phyla	112	111	127	148	161	175	169
Classes	296	327	360	425	488	538	571
Orders	816	917	1,163	1,439	1,624	1840	1,976
Families	1,969	2,282	2,886	3,614	4,264	4870	5,311
Genera	7,372	8,778	12,037	15,342	19,153	23,112	27,326
Species	23,458	30,238	45,555	62,291	80,789	107,235	136,646
**Archaeal taxa**							
Phyla	16	18	19	18	20	19	20
Classes	36	42	47	52	60	64	63
Orders	96	103	116	132	148	166	171
Families	238	276	336	456	508	564	603
Genera	534	650	851	1,344	1,586	1,847	2,079
Species	1,248	1,672	2,339	3,412	4,416	5,869	6,968

* See Parks et al., 2022 for data prior to release R04-RS89 when ANI-species clustering was introduced.

Supplementary Table 2. Number of BioProjects contributing genomes to the NCBI Assembly database.

Year	*No. BioProjects*
2017	6,471
2018	7,253
2019	8,249
2020	8,819
2021	8,150
2022	9,500
2023	9,692
2024*	7,230

* As of September, 2024 when data was retrieved for GTDB R10-RS226

Supplementary Table 3. BioProjects with genome assemblies taxonomically classified as metagenomes.

BioProject	*No. Genomes*	*Taxonomic Assignments*	*Pubmed ID*	*Study*
PRJEB76860	2,153	human gut metagenome	na	Metagenome-assembled genomes of gut microbiome from Estonian population
PRJEB68319	2,131	wastewater metagenome	na	European Longitudinal Sewage
PRJEB83610	1,447	goat gut metagenome	na	Tanzanian goat gut microbiomes adapt to roadside pollutants and environmental stressors
PRJEB62834	954	metagenome	37524802	LakePulse MAG Catalogue
PRJEB81441	499	gut metagenome	na	A metagenomic catalogue of the ruminant gut archaeome
PRJEB88947	462	mouse gut metagenome	39230075	MRGM mouse reference gut metagenome
PRJEB41762	447	soil metagenome	35690846	Kilpisjärvi MAGs
PRJEB67571	389	wastewater metagenome	na	Aalborg West WWTP
PRJEB35770	374	freshwater metagenome	31630686	Freshwater Metagenomes from European Lakes and Reservoirs
PRJEB41001	192	sediment metagenome	na	Community shift of a mixed culture during benzene degradation under nitrate reducing conditions
PRJEB31848	172	metagenome	na	4.5-year tundra warming experiment at the CiPEHR study site in Alaska
PRJEB75483	144	plant metagenome	na	HA Lj-SPHERE
PRJEB37465	118	marine metagenome	na	Southern Ocean Metagenomes
PRJEB35096	98	sediment metagenome	na	Seagrass associated sediment metagenomes
PRJEB34461	87	soil metagenome	na	Metagenomic sequencing of microbial communities from Antarctic soils impacted and no-impacted by marine animals
PRJEB9703	71	human gut metagenome	na	Host-pathobiont-microbiota interactions in undernutrition
PRJEB54903	71	hypersaline lake metagenome	na	Brine and viral community exchange in hypersaline systems
PRJEB41764	70	microbial mat metagenome	na	Pozo Bravo metagenome and metatranscriptome samples
PRJEB65852	66	bovine gut metagenome	na	The role of Asparagopsis Taxiformis in ruminal methanogenesis and modulation of metagenomic functional profiles in vitro
PRJEB64011	64	sediment metagenome	na	New species from methanogenic consortia in microcosms
PRJEB27164	60	hydrothermal vent metagenome	na	Manus Basin inactive chimneys
PRJEB67502	58	marine metagenome	na	Particle-attached (3-10µm) metagenomes from 2018 Helgoland Spring Bloom with PacBio sequencing
PRJEB9169	55	human gut metagenome	na	Gut microbiota and motility
PRJEB77434	48	vaginal metagenome	37271782	Prospective Metagenomic Analysis of the Vaginal Microbiota During Pregnancy
PRJEB20068	47	metagenome	na	Year-round agricultural soil metagenomes
PRJEB46766	37	freshwater metagenome	na	Insight into the microbial diversity of four insulated geothermal springs from Kerguelen Islands through metagenomics
PRJEB12502	34	metagenome	na	Diversity of Lignin and Celluose Degrading Bacteria In North American Forest Soils
PRJEB60262	33	marine metagenome	na	Irinovskoe and Semenov II metagenomes
PRJNA663350	30	human sputum metagenome	na	human sputum metagenome
PRJEB78723	30	lichen metagenome	na	Metagenomic libraries produced from intact thalli of Xanthoria parietina
PRJEB27445	30	saltern metagenome	na	Microbial communities dynamics based on mesocosms experiments in "Es Trenc" solar salterns
PRJNA288562	29	human vaginal or oral	26283357	Temporal and Spatial Variation of the Human

		metagenome		Microbiota During Pregnancy
PRJEB20765	28	soil metagenome	29472560	NGEE Arctic Microbial Communities of Polygonal Grounds
PRJEB36976	28	symbiont metagenome	na	Metagenomics of symbiotic Bathymodiolus mussels from a hybrid zone
PRJEB75750	27	brine metagenome	na	Disturbance experiments on extreme halophilic communities
PRJEB9488	25	human gut metagenome	na	Sialylated glycans promote growth in gnotobiotic models
PRJEB71397	25	marine metagenome	na	Cruise a cross the South Pacific gyre
PRJEB81541	25	human gut metagenome	na	Human Gut Archaea collection from Estonian population
PRJEB43308	23	sediment metagenome	na	Microbial stratification and seasonal dynamics in hypersaline anaerobic sediments of solar salterns
PRJEB53217	23	food fermentation metagenome	na	Hi-C metagenomics of spontaneous fermented beverages
PRJEB88700	22	prokaryotic metagenome	na	Cyanosphere of Nostocaceae GFB15
PRJEB30075	20	plant metagenome	na	Leaf nodule sampling of wild Dioscorea sansibarensis from Madagascar
PRJEB22302	20	sediment or groundwater metagenome	na	Tracking carbon-flow from naphthalene degrading bacteria in coal-tar contaminated surface sediments and groundwater
PRJEB10725	19	soil metagenome	na	Oklahoma and Alaska 1-Year Soil Warming Experiment
PRJEB76410	19	mixed culture metagenome	na	Lab contaminants in mixed cultures
PRJEB14899	18	seawater metagenome	na	Oil spill dispersant strategies and bioremediation efficientcy
PRJEB15554	17	hydrothermal vent metagenome	na	Niche partitioning of diverse sulfur oxidizing bacteria at hydrothermal vents
PRJNA819194	16	gut metagenome	35606844	Kyphosid Ruminant Microbial Biodigestor of Seaweeds
PRJEB49968	15	marine metagenome	na	The microbial community of Tonga plumes and the high abundance of Alcanivorax.
PRJEB27434	12	metagenome	na	Multi-omics of incubated agricultural soils
PRJNA273640	12	hot springs metagenome	na	Viral metagenomes generated to explore viral diversity in low biomass environments
PRJEB42843	11	metagenome	na	Antarctic Snow metagenomes and metabarcoding
PRJEB73309	11	bacterioplankton metagenome	na	Effect of dispersal on the community assembly of aquatic microbe
PRJEB9761	11	metagenome	na	Impacts of Timber Harvesting on Cellulolytic Community of Forest Soils
PRJEB45291	10	hypersaline lake metagenome, brine metagenome	na	Global Metagenomic samples from hypersaline environments
PRJEB71360	10	marine sediment metagenome	na	Acidimicrobiia in coastal marine sediments: abundance, taxonomy and genomic potential
PRJEB42267	9	permafrost metagenome	na	Frozen soil to explore cold-adapted microbial trends and enzymes
PRJEB79635	9	bird metagenome	na	Faecal microbiomes of great tits and blue tits
PRJEB27640	7	indoor metagenome	na	Man-made microbial resistances in built environments
PRJEB21768	7	metagenome	na	Analyzing microbial diversty in groundwater of an artesian borehole in an hard rock aquifer
PRJEB65319	7	metagenome	na	Microbial diversty in groundwater of an artesian borehole in an hard rock aquifer
PRJEB14783	6	jellyfish metagenome	na	Metagenomic microbiome of the jellyfish Cotylorhiza tubercular
PRJEB11362	6	hydrothermal vent	27001712	Heterotrophic belt of diffuse hydrothermal

		metagenome		vents
PRJEB18557	5	metagenome	na	Dietary intervention for an obese child
PRJEB28556	5	metagenome	31624340	Extremophilic nitrite-oxidizing Chloroflexi from Yellowstone hot springs
PRJEB45396	5	metagenome	na	Characterizing Bifidobacterium longum subspecies infantis strains in undernourished Bangladeshi infants and gnotobiotic mice
PRJNA374545	5	mollusc metagenome	na	Mollusc metagenome
PRJNA704462	5	psyllid metagenome	na	Metagenomic assembled genomes from Diaphorina citri in California, USA
PRJEB31310	3	anaerobic digester metagenome	na	Novel syntrophic bacteria in full-scale anaerobic digesters revealed by genome-centric metatranscriptomics
PRJEB33009	3	decomposition metagenome	na	Metabolically-dependent Decomposers
PRJEB37532	3	metagenome	na	Cultivation of novel Nitrolancea species
PRJEB46206	3	subsurface metagenome	na	Groundwater from Mallorca Island
PRJEB36324	3	seawater metagenome	na	Three MAGs of SUP05 cluster (family Thioglobaceae) from deep-sea non-buoyant hydrothermal plumes
PRJEB38353	2	metagenome	na	Reducing HRT to shape the chain elongation reactor microbiota
PRJNA42241	1	hot springs metagenome	na	Yellowstone National Park hot spring microbial community
PRJNA386592	1	invertebrate metagenome	29150518	Molecular characterization of an Endozoicomonas sp.-like associated with mass mortalities of king scallop Pecten maximus L. in the United Kingdom
PRJNA438928	1	freshwater metagenome	na	Studies small meromictic lake Trekhtzetnoe
PRJNA523386	1	wasp metagenome	na	Diachasma alloeum and associated bacteria Genome sequencing
PRJDB7139	1	hot springs metagenome	na	Metagenomic analysis of a thiosulfate-disproportionating enrichment culture
PRJDB5105	1	groundwater metagenome	28885627	16S rRNA gene and metagenomic sequencing analysis of a deep granitic aquifer
PRJNA653494	1	algae metagenome	34080906	Algae metagenome
PRJNA937315	1	human eye metagenome	na	Human eye metagenome
PRJNA645260	1	viral metagenome	na	Global deep-sea sediment virome
PRJNA1200942	1	silage metagenome	na	Maize and lupine biomass isolated bacterial strains
PRJEB10268	1	metagenome	na	Interactions of Nitrospira and heterotrophs
PRJEB10267	1	metagenome	na	Interactions of Nitrospira and heterotrophs
PRJEB11544	1	metagenome	na	Draft genome sequence of Paracoccus aminovorans HPD-2
PRJEB21726	1	metagenome	na	Pesticide degrading bacterial populations on glacier
PRJEB28721	1	mine drainage metagenome	na	Carnoulès Metagenome 8 Metagenomic/Metatranscriptomic Assembly
PRJEB26644	1	invertebrate metagenome	na	Genomics of Ca. Riegeria santandreae
PRJEB40748	1	metagenome	na	A marine coculture of archaeal ammonia and bacterial nitrite oxidizers enriched from the North Sea
PRJEB40426	1	biofilm metagenome	na	Amsterdam canal 16S rRNA gene amplicon and metagenome sequencing
PRJEB36155	1	metagenome	na	Naphthenic acid degrading algal-bacterial community
PRJEB60184	1	marine metagenome	na	Responses to organic pollutants in the tropical Pacific and subtropical Atlantic oceans by pelagic marine bacteria
PRJEB64228	1	freshwater sediment metagenome	na	Enrichment of methanotrophs from urban canals
PRJEB27680	1	hypersaline lake	na	High resilience to sudden dilution of

	metagenome	microbial halophilic communities thriving in solar salterns
